# Muscle Status Response to Oral Nutritional Supplementation in Hemodialysis Patients With Protein Energy Wasting: A Multi-Center Randomized, Open Label-Controlled Trial

**DOI:** 10.3389/fnut.2021.743324

**Published:** 2021-12-10

**Authors:** Sharmela Sahathevan, Tilakavati Karupaiah, Ban-Hock Khor, Birinder Kaur Sadu Singh, Zulfitri Azuan Mat Daud, Enrico Fiaccadori, Alice Sabatino, Karuthan Chinna, Abdul Halim Abdul Gafor, Sunita Bavanandan, Ravindran Visvanathan, Rosnawati Yahya, Zaimi Wahab, Bak-Leong Goh, Zaki Morad, Boon Cheak Bee, Hin Seng Wong

**Affiliations:** ^1^Department of Allied Health Sciences, Faculty of Science, Universiti Tunku Abdul Rahman, Perak, Malaysia; ^2^School of BioSciences, Faculty of Health and Medical Sciences, Taylor's University Lakeside, Selangor, Malaysia; ^3^Faculty of Food Science and Nutrition, Universiti Malaysia Sabah, Sabah, Malaysia; ^4^Department of Pharmacy, Hospital Canselor Tuanku Muhriz, Universiti Kebangsaan Malaysia, Kuala Lumpur, Malaysia; ^5^Department of Dietetics, Faculty of Medicine and Health Sciences, Universiti Putra Malaysia, Selangor, Malaysia; ^6^Nephrology Unit, Department of Medicine and Surgery, Parma University Hospital, Parma, Italy; ^7^School of Medicine, Faculty of Health and Medical Sciences, Taylor's University Lakeside, Selangor, Malaysia; ^8^Department of Medicine, Faculty of Medicine, Universiti Kebangsaan Malaysia, Kuala Lumpur, Malaysia; ^9^Department of Nephrology, Kuala Lumpur Hospital, Kuala Lumpur, Malaysia; ^10^Department of Nephrology, Serdang Hospital, Selangor, Malaysia; ^11^National Kidney Foundation, Selangor, Malaysia; ^12^Department of Nephrology, Selayang Hospital, Selangor, Malaysia

**Keywords:** oral nutritional supplementation, nutrition counseling, hemodialysis, protein energy wasting, quadriceps muscle, ultrasound imaging

## Abstract

**Background:** Muscle wasting, observed in patients with end-stage kidney disease and protein energy wasting (PEW), is associated with increased mortality for those on hemodialysis (HD). Oral nutritional supplementation (ONS) and nutrition counseling (NC) are treatment options for PEW but research targeting muscle status, as an outcome metric, is limited.

**Aim:** We compared the effects of combined treatment (ONS + NC) vs. NC alone on muscle status and nutritional parameters in HD patients with PEW.

**Methods:** This multi-center randomized, open label-controlled trial, registered under ClinicalTrials.gov (Identifier no. NCT04789031), recruited 56 HD patients identified with PEW using the International Society of Renal Nutrition and Metabolism criteria. Patients were randomly allocated to intervention (ONS + NC, *n* = 29) and control (NC, *n* = 27) groups. The ONS + NC received commercial renal-specific ONS providing 475 kcal and 21.7 g of protein daily for 6 months. Both groups also received standard NC during the study period. Differences in quadriceps muscle status assessed using ultrasound (US) imaging, arm muscle area and circumference, bio-impedance spectroscopy (BIS), and handgrip strength (HGS) methods were analyzed using the generalized linear model for repeated measures.

**Results:** Muscle indices as per US metrics indicated significance (*p* < 0.001) for group × time interaction only in the ONS + NC group, with increases by 8.3 and 7.7% for quadriceps muscle thickness and 4.5% for cross-sectional area (all *p* < 0.05). This effect was not observed for arm muscle area and circumference, BIS metrics and HGS in both the groups. ONS + NC compared to NC demonstrated increased dry weight (*p* = 0.039), mid-thigh girth (*p* = 0.004), serum prealbumin (*p* = 0.005), normalized protein catabolic rate (*p* = 0.025), and dietary intakes (*p* < 0.001), along with lower malnutrition–inflammation score (MIS) (*p* = 0.041). At the end of the study, lesser patients in the ONS + NC group were diagnosed with PEW (24.1%, *p* = 0.008) as they had achieved dietary adequacy with ONS provision.

**Conclusion:** Combination of ONS with NC was effective in treating PEW and contributed to a gain in the muscle status as assessed by the US, suggesting that the treatment for PEW requires nutritional optimization *via* ONS.

## Introduction

Treating underlying muscle wasting in malnourished patients with chronic kidney disease (CKD) is challenging. The onset of muscle wasting establishes at the early stages of CKD, and the commencement of the dialysis treatment at end-stage kidney disease is an iatrogenic factor for malnutrition as it also promotes muscle proteolysis (1). The issue of muscle wasting is associated with protein energy wasting (PEW) in patients who undergo dialysis, a syndrome affecting 28–54% of patients worldwide (2). Increased risk for muscle wasting occurs in the presence of uremia, metabolic acidosis, inflammation, and insulin resistance, since each condition promotes muscle proteolysis (1). In patients who undergo dialysis, low muscle mass is associated with frailty, depression, malnutrition (3), and poor quality of life (4–6); and is a strong predictor of hospitalization and mortality (7, 8). The patients who undergo dialysis and having greater muscle mass achieve better physical ability, quality of life and survival (7, 9), indicating a priority to target treatment to mitigate muscle wasting.

Nutritional supplementation and exercise training are recommended to treat muscle wasting in patients with hemodialysis (HD) (6). However, the evidence of benefit for muscle status is inconclusive depending on treatment duration, feeding frequency, nutrient composition of supplementation, the severity of malnutrition, as well as assessment parameters (10, 11). Anomalies arise from malnutrition diagnosis that adopts body mass index (BMI) and serum albumin cut-offs, as muscle and fat compartments of the body are not differentiated, and albumin values are influenced by the presence of inflammation (12, 13). In contrast, although diagnosis with composite nutritional indices, such as subjective global assessment and malnutrition-inflammation score (MIS) (14–16) indicate that oral nutritional supplementation (ONS) improved the overall nutritional status, but muscle mass improvement could not be ascertained. In addition, anomalies in muscle mass assessment arise from sensitivity to detect change as per muscle indices (11) and site of measurement (17) when using skinfold measurements (15), handgrip strength (HGS) (18), and bio-impedance spectroscopy (BIS) (19).

Till now, ONS investigations addressing muscle wasting in patients with HD reflect either ONS use alone (15, 19, 20) or in combination with exercise (11, 18, 21). Studies on ONS intervention alone found no changes in lean body mass (LBM) (15, 19, 20). Exercise therapy alone did not find any improvement in LBM but was associated with improvements in the physical functioning and quality of life (18). In studies evaluating ONS combined with resistance exercise, no significant gains in LBM were apparent (18, 21). These studies investigating exercise alone or in combination with ONS, report no improvement in muscle mass with both treatments. Additionally, these studies recruited patients with mild to severe malnutrition defined by BMI, arm circumference, serum albumin, dietary intake, and subjective global assessment scores (15–17, 20), thus highlighting recruitment without PEW diagnostic criteria.

Assessment of ONS as a treatment strategy in patients with established PEW lacks in terms of the limitation of the methods to indicate improvement in muscle mass. These studies have not adopted direct measurement of muscle mass quantification of the lower limb muscle, which is sensitive to degradation from inflammation-related malnutrition (22). Additionally, there has been no standardization in diagnosing PEW. We addressed these gaps by purposively selecting only HD patients with PEW as the study population and provided them intervention in the form of ONS combined with NC or NC alone. The outcome measure resulted in the change in the muscle status as assessed using ultrasound (US) imaging as per the thickness of the quadriceps muscle and its cross-sectional area (23). PEW was identified in a HD population using the diagnostic criteria of the International Society of Renal Nutrtition and Metabolism (ISRNM) (14). The aims of this study therefore were (i) to assess muscle status changes in response to treatments using the US method and (ii) to determine PEW prevalence post-intervention between the treatment groups.

## Materials and Methods

### Study Design and Patient Recruitment

This multi-center, randomized open-label controlled trial was conducted between June 2016 and July 2019 with recruitment from 16 outpatient HD facilities representing government, private, and non-governmental organization sectors in the Klang Valley. Since serum prealbumin is a stable biomarker for protein synthesis in line with anabolism (24), the sample size calculation was therefore based on the study by Malgorzewicz et al. (16) who used serum prealbumin as an endpoint to ONS provision. In our calculation, using the mean difference in serum prealbumin at 0.57 ± 8.1 g/L, the effect size calculated at 0.70 (moderate effect) with power at 80% and the level of significance set at 5%, the minimum required sample size was 25 patients per treatment arm. Assuming a 20% dropout, the final sample size was inflated to 30 patients per arm.

Eligibility criteria included HD patients receiving standard dialysis treatment (3 sessions per week, 4 h per session) for ≥3 months, aged between 18 and 70 years old, and diagnosed with PEW using the ISRNM criteria (13). The PEW was identified when any 3 out of 4 ISRNM diagnostic criteria were met: BMI <23 kg/m^2^, reduction >10% in MAMC related to the 50th percentile of the reference population, serum albumin <38 g/L, and dietary energy intake (DEI) <25 kcal/kg ideal body weight (IBW).

Patients with a history of poor adherence to HD treatment, prolonged hospitalization, or surgery in the past 3 months prior to recruitment, diagnosed with inflammatory diseases or malignancy, vegetarian, or on regular ONS were excluded.

The study was approved by the Medical Research and Ethics Committee, Ministry of Health, Malaysia (NMRR-16-2525-32068) and the Research Ethics Committee of National University of Malaysia (NN-081-2016). This trial was also registered on www.clinicaltrials.gov (NCT04789031).

### Intervention and Control Groups

The treatments provided were ONS and nutrition counseling (NC). The selected ONS was a renal-specific product (Novasource^TM^ Renal; Nestle Health Science, Malaysia) providing 475 kcal and 21.7 g of protein per serving given on a daily basis. This was a ready-to-drink formula available as a 237 ml tetrabrik pack. The selected product fulfilled the criteria of been calorie and protein dense within a limited volume of supplement, which enables in achieving the necessary nutrient adequacy to improve the nutritional status as well as avoid overhydration in patients with HD. Patients were advised to consume the beverage 30 min after commencing their dialysis session on dialysis days and at home on non-dialysis days. The nutritional information of the product is provided in the Supplementary Table S1.

Nutrition counseling was provided to both the treatment groups by dietitians who counseled on achieving nutritional adequacies for energy and protein whilst limiting sodium, phosphate, potassium, and fluid intakes as per the Kidney Disease Outcomes Quality Initiative Guidelines (25). NC sessions were organized at baseline, third, and sixth months of the study as per standard healthcare protocol.

Recruited patients were randomized in a ratio of 1:1 to receive either ONS + NC or NC only. The NC only group served as the control. Block randomization was carried out at each study site using a computerized randomization calculator (Random Allocation Software Version 1.0) after the baseline data was collected. Randomization was performed by the study statistician (KC), who was not clinically involved in the trial. Both the groups were matched for age, gender, serum pre-albumin, and BMI. Both the groups received treatment for 6 months.

### Outcome Measures

Evaluation of nutritional outcomes was performed at baseline, third, and sixth months of the study and related to the following parameters:

#### Muscle Indices

**Quadriceps muscles:** The thickness of the mid-length (MID) of quadriceps muscle, *rectus femoris* (RF_MID_) and *vastus intermedius* (VI_MID_) muscles, and cross-sectional area (CSA) of the RF (RF_CSA_) at the mid-thigh were assessed using a portable US imaging device (GE Logiq e Digital Portable Color Doppler, GE Healthcare, Wauwatosa, USA). Only one leg was consistently measured for all timed events, with selection for each patient dependent on either the dominant leg or leg without the vascular access. Standardized anatomical landmarking was performed at the MID site as per the International Society for Advancement of Kinanthropometry (ISAK) protocol (26) by an ISAK-trained anthropometrist (TK) as detailed previously (27). Two US scan readings were obtained for each measured site, and the mean value was used for data analysis. Researchers (SS and BHK) performed the US scan 2 h after the commencement of dialysis, with dialysis chairs adjusted for the supine position and both knees extended but relaxed. The same assessor performed all measurements for the same patient throughout the study. The intra- and inter-observer reliability for US measurements has been reported elsewhere (27).

**Arm muscles:** In order to determine the mid-arm muscle circumference (MAMC) and mid-arm muscle area (MAMA) (28), the skinfold thickness and the MAMC of triceps were measured according to the ISAK protocol on the dominant or non-fistula arm (26) using the Harpenden skinfold caliper (HSK-BI; British Indicators, West Sussex, UK) and a no-stretchable tape (Lufkin^®^, Apex Tool Group, LLC, NC, USA). All measurements were collected before the commencement of the dialysis by the same dietitian (SS) to minimize inter-observer variation.

**Bio-impedance spectroscopy (BIS) analysis:** Body composition was assessed using a portable whole-body BIS device (Body Composition Monitor, Fresenius Medical Care, Bad Homburg, Germany) before the dialysis session on a mid-week day, with the patient resting in the supine position. Hydration status, lean tissue mass (LTM), LTM corrected for height (lean tissue index, LTI), and body cell mass (BCM) data generated were based on the physiological tissue model (29).

**Handgrip strength (HGS) test:** HGS was assessed using a digital hand dynamometer (Jamar^®^ Plus +, Sammons Preston, Illinois, USA) on the dominant or non-fistula hand, in a standing position with the arm held straight, at 90° to the trunk of the body (30). The median of the three readings was taken. All measurements were collected before the commencement of dialysis.

#### Diagnosis of Malnutrition

**Malnutrition-inflammation score (MIS) evaluation:** The MIS form was used to assess the severity of the malnutrition-inflammation complex syndrome (31). This fully quantitative nutrition screening tool assessed the domains of weight changes, dietary intake, gastrointestinal system, functional capacity, presence of comorbidities, presence of muscle, and fat depletion as well as BMI, serum albumin, and total iron-binding capacity. The cumulative score for MIS ranges between 0 (normal) and 30 (severely malnourished).

#### Other Nutritional Indicators

- Postdialysis weight was measured using a digital scale (SECA, Model 220, SECA, Germany). This weight was used to calculate the BMI based on the Quetelet's Index (32).
- The mid-thigh girth measurement was taken at the mid-point of the same leg as assessed for the quadriceps, following the ISAK protocol (26).
- Laboratory measures for serum albumin (bromocresol green method), serum prealbumin (immunoturbidimetric method), and high-sensitivity C-reactive protein (hsCRP) (particle-enhanced immunoturbidimetric assay) were analyzed by an accredited external laboratory (Clinipath Sdn Bhd). Interleukin-6 (IL-6) was measured by the sandwich enzyme-linked immunosorbent assay method in our laboratory (SSN and SS) using a commercial kit, IL-6 High Sensitivity Human ELISA kit (Abcam, Cambridge, MA, USA). All biochemistry analyses were based on the mid-week collection of fasting blood samples.
- The appetite of the patient was assessed using the first question from the original 44-item Appetite and Dietary Assessment Tool used in the Hemodialysis Study Group study (33). It was a single, self-administered question with multiple-choice responses: *During the past week (7 days), how would you rate your appetite?* Patients were required to indicate their responses using a scale of 1–5: (1) very good, (2) good, (3) fair, (4) poor, or (5) very poor. Obtained ratings were further classified as “good” (*very good* and *good*) or “diminished” (*fair, poor*, and *very poor*) appetite.
- Physical activity level (PAL) was assessed using an interviewer-administered International Physical Activity Questionnaire (34), which included activities, such as walking, moderate- and vigorous-intensity activities. Scores were expressed in MET-min/week, whereby a minimum of 600 MET-min was identified as moderate-active.

### Monitoring Parameters

**Dietary assessment:** Twenty-four-hour dietary recalls were collected for 3 days, inclusive of a dialysis day, a non-dialysis day, and a weekend (25). This was an interviewer-administered questionnaire with familiarization of household measurements to assist patients in quantifying their dietary intake. Energy and protein intakes were analyzed using Nutritionist Pro™ 2.2.16 software (First DataBank Inc., 2004).

**Clinical parameters:** Normalized protein catabolic rate (*n*PCR), an indirect measure of dietary protein intake (35), was calculated by the participating dialysis centers using an online urea kinetic modeling calculator (36). Dialysis adequacy (Kt/V) was also calculated using the same approach (36). Levels of routine biochemistry parameters, analyzed in-house, such as serum urea, creatinine, and phosphate were obtained from the medical record of the patients.

### Compliance and Product Acceptance

Dialysis nurses at respective study sites ensured that the patients ingested the supplement in their presence on dialysis days. Patients receiving ONS were replenished with a biweekly supply of supplements for home consumption on non-dialysis days. Empty tetrabrik packs or unused supplements were collected biweekly by the researcher to record and monitor the actual intake of ONS. Patients with consecutive ONS intakes <50% during the first 3 months of supplementation were classified as non-compliant.

Patients also fulfilled a product acceptance form (32, 37) on a three-monthly basis. Using a 5-point *Likert* scale, patients evaluated the ONS product based on taste, odor, and portion provided, and rated their overall liking toward the product.

### Charlson Comorbidity Index

The Charlson Comorbidity Index was computed using 19 comorbid conditions, which were weighted and summed to an index on a 0–33 scale (38).

### Statistical Analysis

“Per protocol” analysis was used to exclude patients who either withdrew their participation from the study or those who were non-compliant. Variables were presented as mean ± SD, median (interquartile) or frequency (percentage). The normal distribution of continuous variables was assessed using Kolmogorov–Smirnov test. Continuous variables were analyzed using Student's *t*-test, whereas categorical variables were evaluated using Chi-square test. The group × time interaction on all outcome measures were analyzed using a generalized linear model for repeated measures, with Bonferroni *post-hoc* test. Univariate analysis was used to compare percentage change between groups. All analyses were computed using the IBM Statistical Package for Social Sciences version 26.0 (IBM SPSS Statistics Inc. Chicago IL. USA). Statistical significance was set at *p* < 0.05 for all evaluated parameters.

## Results

### Study Stock Flow

**Figure 1:**
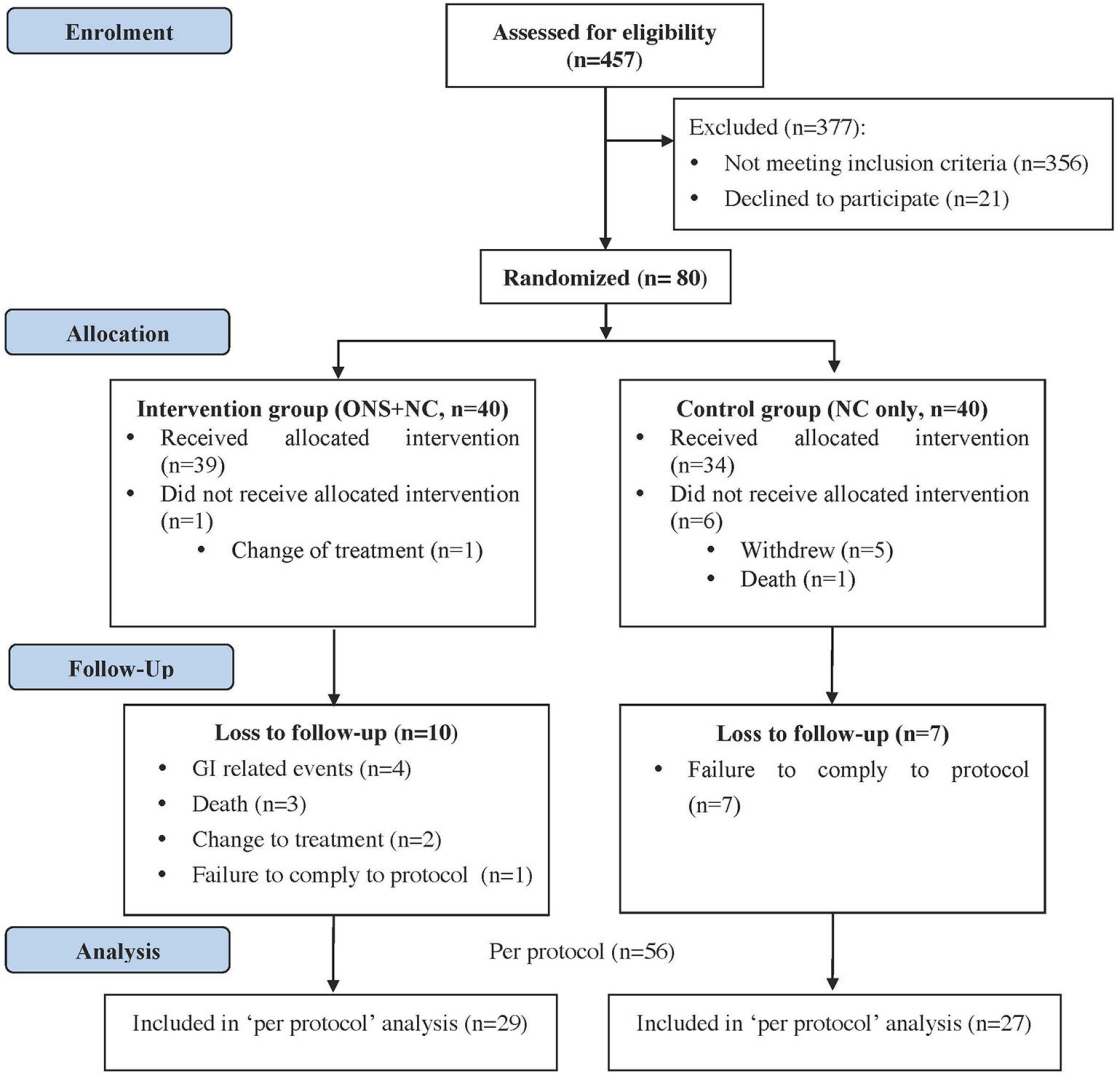
Study flow diagram.

Out of 101 eligible HD patients identified with PEW, only 80 patients consented to participate in this study. Upon randomization, 40 patients were allocated to each treatment arm. The stock flow of patients according to the Consort diagram is presented in Figure 1.

Study withdrawals after randomization and consent giving occurred at baseline from patients withdrawing consent (NC, *n* = 5) or who became eligible for transplant (ONS + NC, *n* = 1). One death from a cardiac event (NC) occurred before the initiation of the study. The dropout rate at the end of the intervention was 30%.

### Baseline Characteristics of Patients

Baseline characteristics of patients completing the protocol are shown in Table 1. Age, gender, dialysis vintage, the Charlson comorbidity index, comorbidities, dialysis adequacy, and type of vascular access were not significantly different between groups except for the presence of diabetes mellitus (*p* = 0.023). Similarly, no difference in nutritional status and PAL was observed between both groups.

**Table 1:** Baseline characteristics of study patients.

**Patient characteristics**	**ONS + NC** **(***n*** = 29)**	**NC** **(***n*** = 27)**	***P*** **-value**
Age (years)^a^	50.90 ± 11.41	48.85 ± 15.97	0.582
Gender (male/female)	17/12	18/9	0.534
Dialysis vintage (months)	91 ± 85	61 ± 53	0.130
Charlson comorbidity index	4.17 ± 1.49	4.52 ± 2.01	0.465
**Co-morbidities** **(***n***, %)**^b^			
Diabetes	4 (13.8)	11 (40.7)	0.023
Hypertension	20 (69.0)	21 (77.8)	0.457
Hepatitis B or C	5 (17.2)	5 (18.5)	0.587
Cardiovascular disease	2 (6.9%)	3 (11.1%)	0.465
Kt/V	1.73 ± 0.46	1.76 ± 0.32	0.790
**Vascular access** **(***n***, %)**			
Fistula	24 (82.8%)	21 (77.8%)	0.636
Catheter	5 (17.2%)	6 (22.2%)	
**Nutritional parameters**			
BMI (kg/m^2^)	19.85 ± 2.00	19.83 ± 2.49	0.984
MAMC (cm^2^)	21.05 ± 2.32	21.14 ± 2.33	0.895
Serum albumin (g/L)	41.83 ± 3.71	41.85 ± 3.12	0.984
Serum prealbumin (g/L)	0.28 ± 0.09	0.26 ± 0.07	0.386
DEI (kcal/kg IBW)	25.21 ± 7.03	24.14 ± 6.01	0.546
MIS score	7.41 ± 2.77	6.19 ± 2.95	0.114
PAL (MET-minutes/week)	198 (0–487)	198 (0–396)	0.980
Hydration status (kg)	2.46	2.62	0.737

a *Continuous data were analyzed using Students t-test and presented as mean ± SD or median (interquartile)*.

b *Categorical data were analyzed using Chi-square test and presented as frequency (percentage)*.

*BMI, body mass index; DEI, dietary energy intake; IBW, ideal body weight; MAMC, mid-arm muscle circumference; MIS, malnutrition-inflammation score; NC, nutrition counseling; PAL, physical activity level; Kt/V, dialysis adequacy; ONS, oral nutritional supplementation*.

### Muscle Status

#### Ultrasound-Derived Muscle Metrics

**Figure 2:**
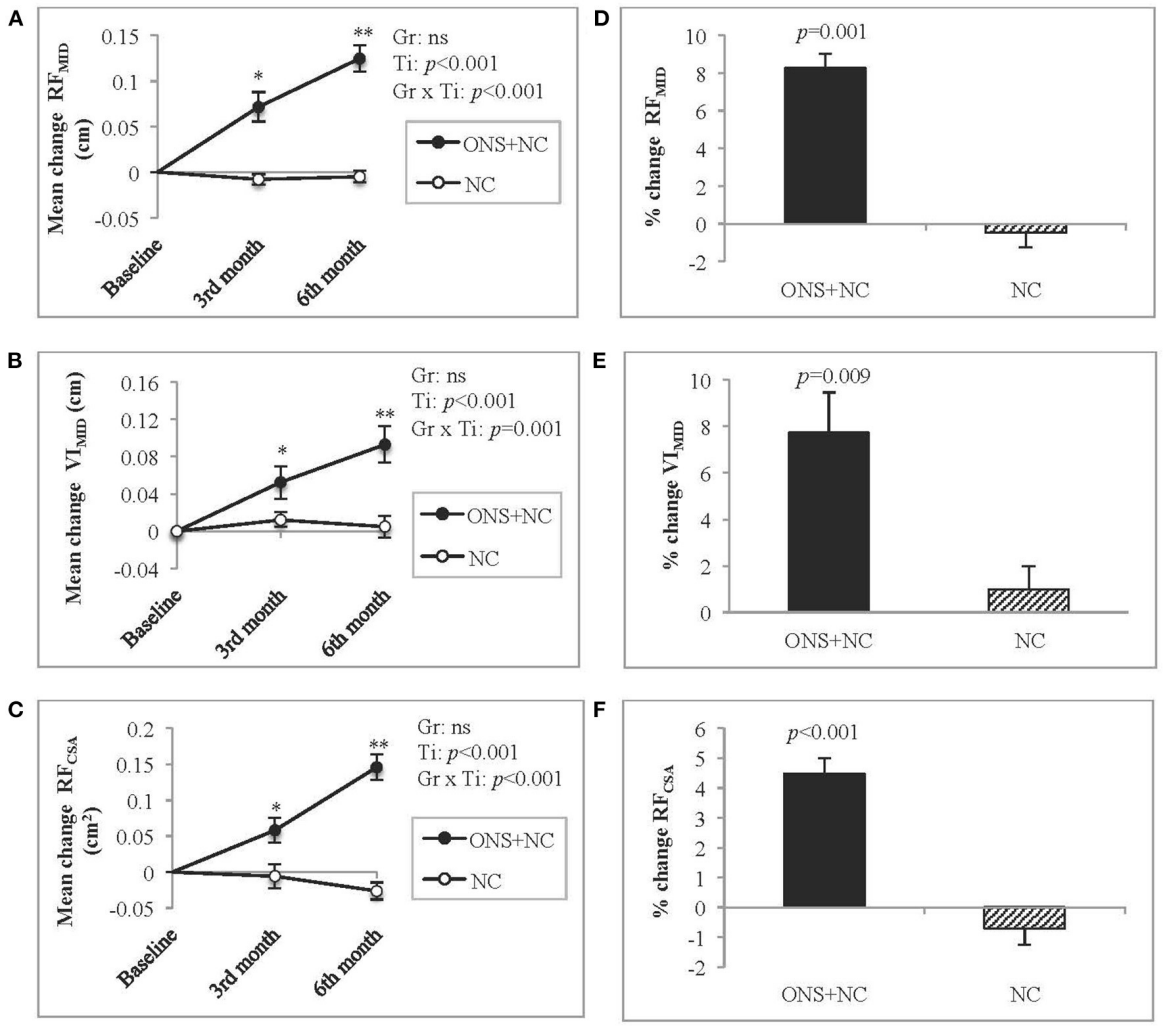
Changes in US-derived muscle metrics. **(A–C)** represent mean changes according to US metrics for **(A)** RF_MID_, **(B)** VI_MID_, and **(C)** RF_CSA_, whereas **(D–F)** represent the percentage change for **(D)** RF_MID_, **(E)** VI_MID_, and **(F)** RF_CSA_. Gr, main effect of Group; Ti, main effect of Time, Gr × Ti, Group × Time interaction; ^*^p < 0.05; ^**^p < 0.001; Data were adjusted for age, gender, dialysis vintage, and presence of diabetes mellitus. CSA, cross-sectional area; MID, mid-point; NC, nutrition counseling; ns, not significant; ONS, oral nutritional supplementation; RF; *rectus femoris*; US; ultrasound; VI, *vastus intermedius*.

Mean changes according to treatment response (group × time interactions) for US-derived muscle metrics are provided in the Supplementary Table S2. The group × time interactions were significant for RF_MID_, VI_MID_, and RF_CSA_ (Figures 2A–C). Increasing trends in all US metrics observed only in the ONS + NC group, were significant for mean changes from baseline to 3rd and 6th months of the study. The NC group experienced no change to these metrics. ONS + NC patients also experienced significant increases of 8.3% for RF_MID_ (*p* = 0.001), 7.7% for VI_MID_ (*p* = 0.009), and 4.5% for RF_CSA_ (*p* < 0.001) compared to minimal changes in these metrics in the NC group (Figures 2D–F).

#### Other Muscle Indices

Treatment responses for other muscle indices are shown in Table 2. Group × time interactions were not significant for MAMC, MAMA, BIS-derived muscle measures, and HGS (all *p* > 0.05). However, a significant increase over time was detected at the 3rd month for ONS + NC treatment as per MAMC (*p* < 0.001) and MAMA (*p* < 0.001), but this effect was not apparent at the 6th month. The NC treatment effected no change for these measures.

**Table 2:** Changes in other muscle indices according to treatment groups.

	**ONS + NC** **(***n*** = 29)**					**NC** **(***n*** = 27)**				
	**Baseline**	**3rd month**	**6th month**	**Δ** ^***t3***^	**Δ** ^***t6***^	**Baseline**	**3rd month**	**6th month**	**Δ** ^***t3***^	**Δ** ^***t6***^
**Arm circumference**										
MAMC (cm^2^)^b^	20.4 ± 0.5	20.9 ± 0.5	20.8 ± 0.5	0.5 ± 0.1^**^	0.4 ± 0.2	20.6 ± 0.4	20.9 ± 0.4	20.8 ± 0.3	0.3 ± 0.2	0.2 ± 0.3
MAMA (cm^2^)^b^	24.9 ± 1.5	26.8 ± 1.5	26.4 ± 1.6	1.9 ± 0.4^**^	1.4 ± 0.6	25.9 ± 1.2	26.8 ± 1.2	26.2 ± 1.1	1.0 ± 0.6	0.3 ± 0.9
**Body composition**									
LTM (kg)	29.2 ± 1.3	29.5 ± 1.3	29.4 ± 1.3	0.3 ± 0.3	0.2 ± 0.4	28.6 ± 1.0	28.6 ± 0.9	28.5 ± 1.0	−0.1 ± 0.2	−0.1 ± 0.3
LTI (kg/m^2^)	11.4 ± 0.5	11.7 ± 0.5	11.6 ± 0.5	0.3 ± 0.2	0.1 ± 0.2	11.7 ± 0.4	11.8 ± 0.4	11.8 ± 0.4	0.1 ± 0.1	0.2 ± 0.2
BCM (kg)	15.3 ± 1.0	15.5 ± 1.0	15.4 ± 1.0	0.3 ± 0.3	0.1 ± 0.4	15.7 ± 0.8	15.9 ± 0.7	15.9 ± 0.7	0.2 ± 0.3	0.2 ± 0.4
**Muscle strength**										
HGS (kg)	19.0 ± 1.3	19.1 ± 1.3	19.6 ± 1.4	0.1 ± 0.4	0.6 ± 0.4	18.3 ± 0.8	18.6 ± 0.9	19.1 ± 0.9	0.3 ± 0.4	0.8 ± 0.4

*All baseline comparisons between group were not significantly different as per Independent t test; Data adjusted for age, gender, dialysis vintage, and presence of diabetes mellitus are presented as mean ± SD; ^a^Main effect of Group*,

b Main effect of Time, ^c^ Group × Time interaction; Δ^t3^, Mean change at 3rd month; Δ^t6^, Mean change at 6th month; *p < 0.05 compared to baseline,

** *p < 0.001 compared to baseline*.

*BCM, body cell mass; HGS, handgrip strength; LTI, lean tissue index; LTM, lean tissue mass; MAMA, mid-arm muscle area; MAMC, mid-arm muscle circumference; NC, nutrition counseling; ONS, oral nutritional supplementation*.

### Malnutrition Diagnosis

#### MIS Evaluation

**Figure 3:**
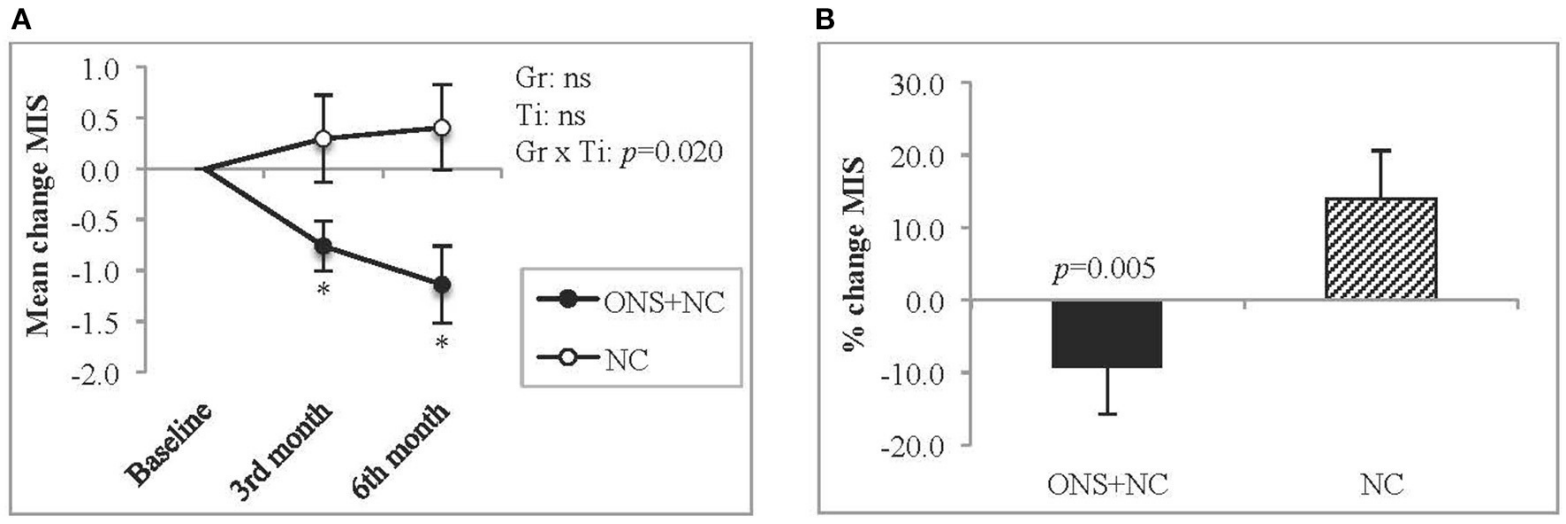
Changes in MIS score. This figure represents **(A)** mean changes and **(B)** percentage change for MIS score. Gr, main effect of Group; Ti, main effect of Time; Gr × Ti, Group × Time interaction; ^*^p < 0.05; Data were adjusted for age, gender, dialysis vintage, and presence of diabetes mellitus. MIS, malnutrition-inflammation score; NC, nutrition counseling; ns, not significant; ONS, oral nutritional supplementation.

The group × time interactions for MIS was significant, with declining trends in ONS + NC groups at both the 3rd (*p* = 0.032) and 6th months (*p* = 0.041) of the study (Figure 3A). In contrast, the NC group experienced no improvement in MIS scores. The percentage change for MIS score in ONS + NC group was significant (−9.4 %, *p* = 0.005) compared to 14% increase in NC group (Figure 3B).

#### PEW Status

Eligibility criteria for PEW diagnosis met by patients were mainly reduced for the BMI (100%), MAMC (ONS + NC = 89.7% vs. NC = 88.9%), and DEI criteria (ONS + NC = 62.1% vs. NC = 77.8%) (all *p* > 0.05) (Table 3). Only a small percentage of patients met the low serum albumin criteria (ONS + NC = 20.7% vs. NC = 18.5%) (*p* > 0.05). By the end of the 6-month treatment, a change in PEW eligibility criteria only occurred as per the DEI criteria with patient numbers reducing in the ONS + NC group compared to the NC group (ONS + NC = 24.1% vs. NC = 70.4%, *p* = 0.001). This resulted in lower PEW prevalence with ONS + NC compared to NC treatment (24.1 vs. 59.3%, *p* = 0.008).

**Table 3:** Effect of treatment on PEW criteria status.

	**Baseline**		***P*** **-value**	**6th month**		***P*** **-value**
**PEW criteria^a^, ^b^**	**ONS + NC** **(***n*** = 29)**	**NC** **(***n*** = 27)**		**ONS + NC** **(***n*** = 29)**	**NC** **(***n*** = 27)**	
BMI <23 kg/m^2^	29 (100%)	27 (100%)	NA	26 (89.7%)	24 (88.9%)	0.630
MAMC >10th percentile	26 (89.7%)	24 (88.9%)	1.000	24 (82.8%)	22 (81.5%)	0.587
Serum albumin <38 g/L	6 (20.7%)	5 (18.5%)	0.838	5 (17.2%)	7 (25.9%)	0.429
DEI <25 kcal/kg IBW	18 (62.1%)	21 (77.8%)	0.201	7 (24.1%)	19 (70.4%)	0.001

a *Categorical data were presented as frequency (percentage)*.

b *Data was analyzed using Chi-square test*.

*BMI, body mass index; DEI, dietary energy intake; IBW, ideal body weight; MAMC, mid-arm muscle circumference; NA, not available; NC, nutrition counseling; ONS, oral nutritional supplementation; PEW, protein energy wasting*.

### Other Nutritional Outcomes

**Figure 4:**
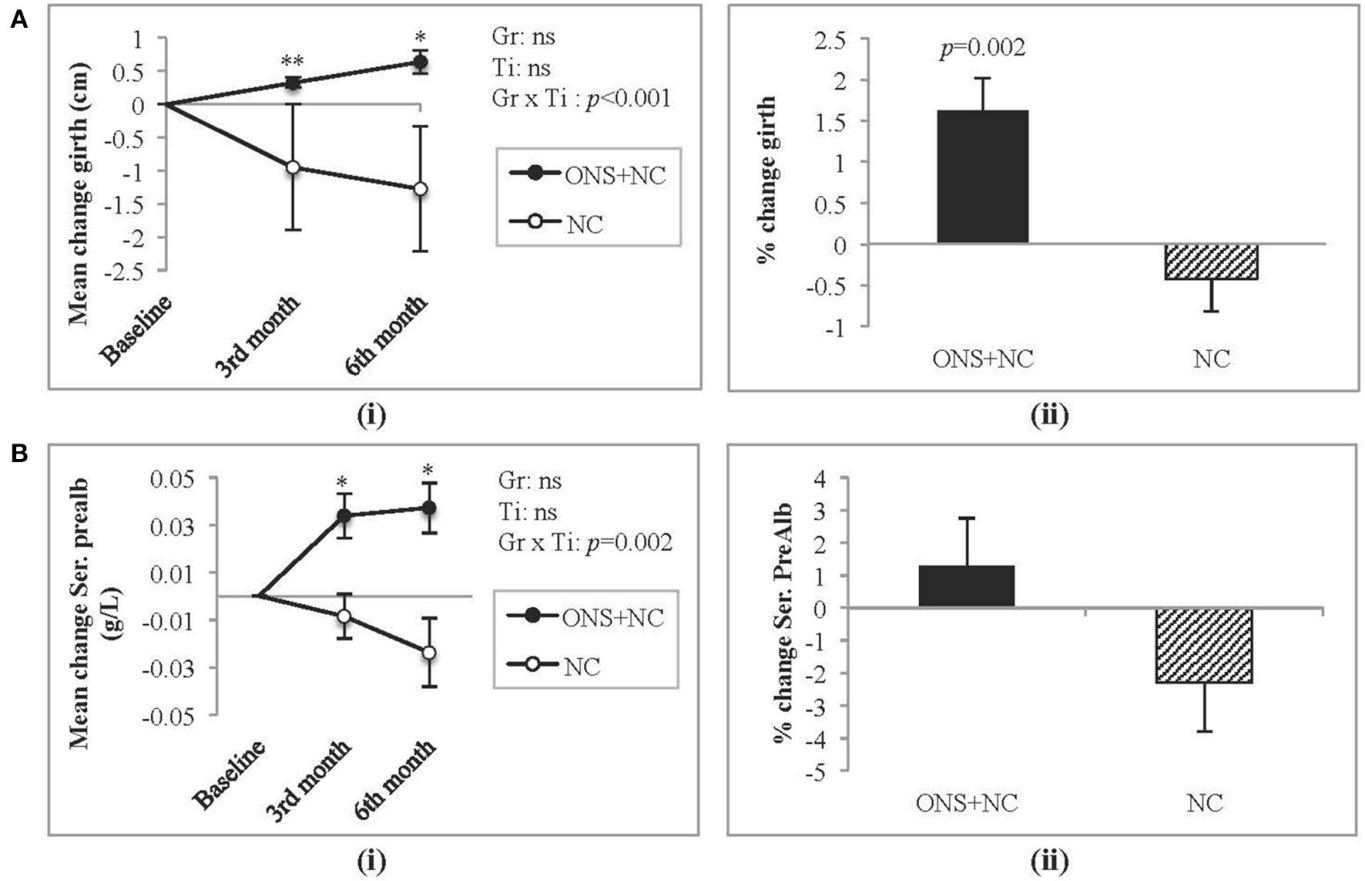
Changes in mid-thigh girth and serum prealbumin levels. **(A)** Represents (i) mean change and (ii) percentage change in mid-thigh girth, whereas **(B)** represents (i) mean change and (ii) percentage change in serum prealbumin. Gr, main effect of Group; Ti, main effect of Time; Gr × Ti, Group × Time interaction; ^*^p < 0.05; ^**^p < 0.001; Data were adjusted for age, gender, dialysis vintage, and presence of diabetes mellitus. NC, nutrition counseling; ns, not significant; ONS, oral nutritional supplementation; Prealb, prealbumin.

Treatment response (group × time interactions) for other nutritional parameters are provided in the Supplementary Table S3. Parameters that were not significantly different between and within treatment groups were BMI, serum albumin, creatinine, phosphate, hsCRP, IL-6, appetite ratings, and PAL. Positive improvements, however, were gained only by the ONC + NC group at 6 months for dry weight (mean change = 1.1 ± 0.4 kg, *p* = 0.039). For this group, specific significant increases in mid-thigh girth (Figure 4A) and prealbumin (Figure 4B) occurred with each time point, resulting in a significant percentage change increase of approximately 2% only for mid-thigh girth. Adequacy with ONS supplementation reflected in improved *n*PCR (mean change = 0.2 ± 0.1 g/kg, *p* = 0.025) and dietary parameters (mean change for energy intake = 366 ± 60 kcal/day, *p* < 0.001; mean change for protein intake =17.4 ± 3.2 g/day, *p* < 0.001).

### Product Monitoring and Acceptance

There was no change in the hydration status or hospitalization frequency over the 6 months of intervention (data not shown). The average compliance rate achieved by patients receiving ONS was 81%. Product acceptance in relation to taste, flavor, and portion size was reported at 90% with minimal (<10%) reporting of dislike toward the odor of the product, satiety, and adverse events for the ONS group.

## Discussion

Treatment strategies toward reducing the progression of muscle wasting are challenging given the complex etiology of PEW, and the current lack of evidence to support ONS or exercise or both options as beneficial in mitigating muscle wasting. The major finding from our study, which recruited only patients with PEW, was that those receiving ONS with nutrition counseling (ONS + NC) demonstrated significant improvement in quadriceps muscle indices, namely RF_MID_, VI_MID_, and RF_CSA_, as measured by the US approach. Additionally, these patients demonstrated improvements in the nutritional status and lower MIS scores with concomitant gains in dry weight, mid-thigh girth, serum prealbumin and *n*PCR. These improvements were not observed in the group receiving only NC.

We detected improvements in muscle status in response to ONS treatment using the US approach. The clinical significance of our data indicates 8.3, 7.7, and 4.5% improvements in RF_MID_, VI_MID_, and RF_CSA_, respectively. In contrast, only one study reported a 4.2% increase in arm muscle circumference in patients with HD supplemented with ONS for 12 weeks (21). We do note that studies reporting US measure for various clinical outcomes in different populations. The thickness of the lower quadriceps muscle predicted fall injury (39) and PEW risk (40) in HD patients. The thickness of the lower quadriceps muscle was also associated with prolonged hospitalization in critically ill patients (41), whereas lower RF_CSA_ had been associated with hospital readmission or death in patients with chronic respiratory disease (42). The advantage of US is that it directly quantifies muscle thickness and CSA (43), allowing for the detection of small changes in muscle status attributed to nutritional intervention (43). Importantly, overhydration status is not an issue for US measurements as consistency of readings for pre- and post-dialysis is reported (44). The usability and low-cost US for muscle status assessment contrasts with gold standard methods, such as dual energy X-ray absorptiometry, magnetic resonance imaging, or computed tomography, which although having high accuracy and validity are not feasible for routine clinical use as they require trained personnel, are costly, and also pose radiation risk to patients with CKD (4, 45, 46). This further justifies the use of the US as an alternative bedside measure as it has been validated against computed tomography (24) to detect muscle wasting in HD patients (40). Interestingly, the NC group did not demonstrate any significant improvement in US measures as experienced by the group receiving ONS. Of note, the absence of deterioration in their muscle status could be attributed to the provision of nutrition counseling.

No change in muscle indices was observed with BIS or HGS assessments as comparator assessments. It should be noted that BIS only provides mathematical estimates of muscle mass (19, 29) and its precision in estimating LTM is affected by hydration status (47). Ideally, the BIS assessment should be performed post-dialysis, as patients are closest to their dry weight (47, 48). This may perhaps explain the lack of significance we observed as the BIS measurement was performed pre-dialysis to suit the convenience of the patients. As regards the non-significant outcomes of HGS measurement reported in this study, weakness arising from poor physical activity, a common scenario in the HD population may contribute to “muscle disuse” (17). Indeed, in an earlier cross-sectional study of US measurement in Malaysia, we noted that there was no difference in HGS between PEW and non-PEW HD patients (40).

We additionally provided MIS evaluation in the assessment monitoring protocol, as the complex milieu of malnutrition-inflammation is implicated in muscle wasting. Malnutrition coexists with inflammation in dialysis patients (31), and inflammation is a contributive factor to malnutrition and poor appetite (2, 49). We found the patients receiving ONS + NC compared to the NC group achieved significantly lower MIS scores by the end of 6 months in tandem with an improvement in nutritional status, although inflammatory markers were not different after treatments. Ko et al. (50) have noted low levels of leptin, an appetite-suppressing hormone associated with proinflammatory properties and that high CRP levels were associated with malnourished patients with HD, who were identified using MIS scores. Comparatively, patients in the present study had lower CRP levels, which is similar to a Japanese HD cohort in the Phase 3 *Dialysis Outcomes and Practice Pattern Study* (51). There is no strong evidence supporting the improvement of inflammation status *via* nutritional intervention (52, 53). Alternatively, treatment strategies targeted at improving dialysis-induced inflammation factors or anti-cytokine therapies could be explored (52).

Prealbumin, a negative acute-phase protein is a biomarker sensitive toward rapid changes in nutritional status (14, 54) due to its shorter half-life compared to serum albumin (~2–3 vs. 20 days). Prealbumin is commonly used in nutritional interventions to indicate response toward treatment (55). In our study, the patients on ONS did achieve a significant increase in prealbumin levels as expected from nutritional interventions (14, 35). We did note a non-significant increase in serum albumin levels in the ONS + NC group, which concurs with other ONS studies (13, 15, 56). However, the magnitude of change in albumin status depends on the duration of ONS feeding (10), severity of hypoalbuminemia (56, 57), chronic inflammation (58) and hydration status (59, 60), not withstanding the pro-inflammatory nature of dialysis treatment (61).

We used the PEW-ISRNM diagnostic criteria to identify PEW (13), as it requires objective assessments of muscle wasting. Combination treatment of ONS with NC was beneficial in patients with PEW, as indicated by a significant decline in PEW prevalence at the end of the 6th month. This effect concurs with other studies treating general malnutrition in HD patients (15, 37, 62). Interventional approaches to treat PEW diagnosed by the ISRNM criteria applied by other researchers indicated some limitations to interpretations. Enrolment of both PEW and non-PEW patients occurred with one study (21), another study failed to report the remission of PEW post-treatment (24), whereas the 3rd study was underpowered (total *n* = 16) and targeted only elderly HD patients (18). Further, whether exercise alone in comparison to combination treatments for HD patients are valid strategies that remain inconclusive, as study design limitations, such as inclusion of young and well-nourished patients (11), suboptimal intensity and duration of exercise (63), small sample size (11, 18) and sensitivity of method in assessing muscle mass, are noted (11). Additionally, aiming for dietary energy sufficiency was not planned in these strategies (63).

In terms of the PEW remission associated with ONS intervention that we reported, more patients achieved dietary adequacy compared to NC alone as per DEI >25 kcal/kg IBW (38.0 vs. 7.4%, *p* = 0.001). This indicated that ONS treatment was able to optimize dietary adequacy in malnourished HD patients, thereby fulfilling the study objective. However, we note that the nutritional composition of ONS does differ as reported by various studies, depending on the objectives of the outcome. We ourselves found that a protein only supplementation did not correct for energy deficiency in malnourished peritoneal dialysis patients (64). Supplementation studies in HD patients do concur achieving dietary adequacy for both energy and protein intake were met *via* ONS (11, 21, 65) but not with protein only supplementation (66, 67). Other associated markers of nutritional status that improved with ONS intervention were significant gains in dry weight, mid-thigh girth, serum prealbumin, and *n*PCR levels, which align with dietary adequacy. Nutritional adequacy promotes positive nitrogen balance thus minimizing the catabolic impact of PEW *via* gluconeogenesis (2, 68). In a secondary analysis looking at protein kinetics, Gamboa et al. (69) reported that well-nourished HD patients receiving ONS achieved positive amino acid balance based on increases in their net protein balance in the forearm skeletal muscle.

Poor compliance is a common issue affecting successful ONS intervention (70, 71). Poor compliance (<70%) reported in previous studies were related to taste perceptions, presence of adverse events, and fear of overhydration (70, 71). The dropout rate of 30% in the present study was similar to 31.8% reported by Caglar et al. (70). We avoided the risk of poor compliance in our study by prestesting available renal-specific ONS products in HD patients (*n* = 10) outside the recruitment of this study. This approach was also reported by Patel et al. (56). Additionally, early satiety with ONS intake and reduced dietary intake (37) were avoided by allowing for flexible consumption of ONS between main meals and before going to bed, which allowed patient nutritional intakes to achieve adequacy (37, 71).

The novel finding from this study is improvement in muscle changes in response to ONS treatment, which was detected by the US method, answering the gap in literature for an appropriate impact measure to detect response. Other strengths were using the ISRNM-PEW diagnosis to standardize patient selection criteria, assessing the presence of malnutrition-inflammation complex syndrome as per MIS score, a longer duration of supplementation, and adequate ONS dose. These factors are known to influence the efficacy of ONS in improving the muscle status of HD patients (10, 11, 15). The study methodology adopted only a single leg to measure, so as to minimize technical error of measurement that are likely to happen in the intervention studies.

A major limitation of this study was the unequal distribution of patients with diabetes mellitus between treatment groups. Although the data were adjusted for the presence of diabetes mellitus, it should be noted that diabetes mellitus is the main cause of CKD (71) and insulin resistance is contributive to muscle wasting in HD patients (1, 72). The 6-month study duration was insufficient to measure the impact of muscle change on clinical endpoints as regards to infection rates, hospitalization, and mortality. Furthermore, taste fatigue, a common issue when patients are consuming ONS daily for a prolonged period may have hindered a greater compliance (73, 74) despite the 81% achievement in the current study.

In conclusion, gains in quadriceps muscle status detected using the US approach in patients with PEW on HD were attributed to dietary optimization *via* the ONS provision. A significant reduction in PEW prevalence occurred with the ONS intervention by patients achieving dietary adequacy. Nutritional interventions for the treatment of muscle wasting associated with malnutrition should consider the US approach to monitor the outcomes for clinical relevance.

## Data Availability Statement

The original contributions presented in the study are included in the article/Supplementary Materials, further inquiries can be directed to the corresponding authors.

## Ethics Statement

The studies involving human participants were reviewed and approved by the Medical Research and Ethics Committee, Ministry of Health, Malaysia (NMRR-16-2525-32068) and the Research Ethics Committee of National University of Malaysia (NN-081-2016). This trial was also registered on www.clinicaltrials.gov (NCT04789031). The patients/participants provided their written informed consent to participate in this study.

## Author Contributions

SS and TK designed the study. SS was the main author of the manuscript, performed all the assessment of nutritional outcomes, analyzed, and interpreted data. TK supervised the project. B-HK and BKSS assisted in performing the nutritional assessments. KC assisted with the statistical analysis. SS, TK, B-HK, ZAMD, EF, AS, AHAG, SB, RY, RV, ZW, B-LG, ZM, BCB, and HSW and assisted in the interpretation of the results and writing the manuscript. All authors contributed to the article and approved the submitted manuscript.

## Funding

This research was funded by the National Kidney Foundation, NKF 1001/ADM/753.

## Conflict of Interest

The authors declare that the research was conducted in the absence of any commercial or financial relationships that could be construed as a potential conflict of interest.

## Publisher's Note

All claims expressed in this article are solely those of the authors and do not necessarily represent those of their affiliated organizations, or those of the publisher, the editors and the reviewers. Any product that may be evaluated in this article, or claim that may be made by its manufacturer, is not guaranteed or endorsed by the publisher.
