# Supplementary material for: Muscle Status Response to Oral Nutritional Supplementation in Hemodialysis Patients With Protein Energy Wasting: A Multi-Center Randomized, Open Label-Controlled Trial
Source: Front Nutr. 2021 Dec 10;8:743324. doi: 10.3389/fnut.2021.743324 (PMC8717812; doi:10.3389/fnut.2021.743324)
Supplement: Supplementary file 1 [file Table_1.docx]

Supplementary Material

# Supplementary Tables

Table S1. Characteristic of Novasource Renal

| Characteristic | Description |
| --- | --- |
| Product type | Liquid |
| Volume | 237 ml |
| Calorie/serving | 475 kcal /2 kcal/ml |
| Protein/serving | 21.7 g |
| Fat/ serving | 23.8 g |
| Carbohydrate/serving | 43.5 g |
| Sodium/serving | 225 mg |
| Potassium/serving | 225 mg |
| Phosphate/serving | 195 mg |
| Source of protein | Sodium and calcium caseinates, L-arginine |

Table S2. Changes in US-derived muscle measures according to treatment groups

|  | ONS+NC (n=29) | | | | | NC (n=27) | | | | |
| --- | --- | --- | --- | --- | --- | --- | --- | --- | --- | --- |
|  | Baseline | 3^rd^ month | 6^th^ month | Δ*^t3^* | Δ*^t6^* | Baseline | 3^rd^ month | 6^th^ month | Δ*^t3^* | Δ*^t6^* |
| ***US measures*** | | | | | | | | | | |
| RF_MID_ (cm)*^b,c^* | 1.56 ± 0.1 | 1.63 ± 0.1 | 1.68 ± 0.1 | 0.07 ± 0.0^**^ | 0.12 ± 0.0^**^ | 1.63 ± 0.1 | 1.63 ± 0.1 | 1.63 ± 0.1 | -0.01 ± 0.0 | -0.01 ± 0.0 |
| VI_MID_ (cm)*^b,c^* | 1.39 ± 0.1 | 1.34 ± 0.1 | 1.43 ± 0.1 | 0.05 ± 0.0* | 0.09 ± 0.0^**^ | 1.18 ± 0.1 | 1.19 ± 0.1 | 1.19 ± 0.1 | 0.01 ± 0.0 | 0.01 ± 0.0 |
| RF_CSA_ (cm^2^) *^b,c^* | 5.50 ± 0.3 | 5.56 ± 0.3 | 5.64 ± 0.3 | 0.06 ± 0.0^*^ | 0.15 ± 0.0^*^*^*^* | 5.34 ± 0.2 | 5.33 ± 0.2 | 5.31 ± 0.2 | -0.01 ± 0.0 | -0.03 ± 0.0 |

Note: All baseline comparisons between group were not significantly different as per Independent *t* test; Data adjusted for age, gender, dialysis vintage and presence of diabetes mellitus is presented as mean ± standard deviation; *^a^* Main effect of Group, *^b^* Main effect of Time, *^c^* Group x Time interaction; Δ*^t3^* =Mean change at 3^rd^ months, Δ*^t6^* =Mean change at 6^th^ months; Significance for mean change differences was determined using the Bonferroni post-hoc test with **p*<0.05 compared to baseline and ** *p*<0.001 compared to baseline

Abbreviations: CSA, cross-sectional area; MID, mid-point; NC= nutrition counseling; ONS= oral nutritional supplementation; RF, *rectus femoris*; US, ultrasound; VI, *vastus intermedius*

Table S3. Nutritional outcomes according to treatment groups

| Nutritional outcomes | ONS+NC (n=29) | | | | | | | | NC (n=27) | | | | |
| --- | --- | --- | --- | --- | --- | --- | --- | --- | --- | --- | --- | --- | --- |
|  | Baseline | 3^rd^ month | | 6^th^ month | | Δ*^t3^* | | Δ*^t6^* | Baseline | 3^rd^ month | 6^th^ month | Δ*^t3^* | Δ*^t6^* |
| ***Physical measures*** | | | | | | | | | | | | | |
| Post-dialysis weight (kg)*^b^* | 50.4 ± 1.2 | 51.1 ± 1.2 | | 51.5 ± 1.2 | | 0.7 ± 0.4 | | 1.1 ± 0.4 | 49.6 ± 1.3 | 49.9 ± 1.3 | 50.0 ± 1.2 | 0.3 ± 0.3 | 0.5 ± 0.4 |
| Dry weight (kg)*^b^* | 49.4 ± 1.3 | 50.1 ± 1.3 | | 50.5 ± 1.3 | | 0.7 ± 0.3 | | 1.1 ± 0.4*^*^* | 48.9 ± 1.2 | 49.4 ± 1.2 | 49.5 ± 1.2 | 0.5 ± 0.3 | 0.7 ± 0.4 |
| BMI (kg/m^2^)*^b^* | 20.0 ± 0.4 | 20.2 ± 0.4 | | 20.4 ± 0.4 | | 0.3 ± 0.1 | | 0.4 ± 0.2 | 19.9 ± 0.5 | 20.0 ± 0.5 | 20.0 ± 0.5 | 0.1 ± 0.1 | 0.2 ± 0.1 |
| ***Biochemistry markers*** | | | | | | | | | | | | | |
| Serum albumin (g/L) | 40.8 ± 0.8 | 41.5 ± 1.0 | | 41.5 ± 1.0 | | 0.7 ± 0.6 | | 0.6 ± 0.6 | 41.7 ± 0.6 | 40.6 ± 0.7 | 40.5 ± 0.8 | -1.0 ± 0.5 | -1.2 ± 0.6 |
| Serum urea (mmol/L)*^a,c^* | 19.2 ± 1.1 | 22.7 ± 1.2 | | 21.6 ± 1.2 | | 3.5 ± 1.0*^*^* | | 2.5 ± 0.9 | 19.0 ± 1.2 | 18.4 ± 1.2 | 18.7 ± 1.2 | -0.6 ± 0.8 | -0.3 ± 0.9 |
| Serum creatinine  (μmol/L) | 788 ± 43 | 826 ± 37 | | 826 ± 49 | | 38 ± 22 | | 38 ± 48 | 770 ± 31 | 803 ± 33 | 778 ± 33 | 33 ± 20 | 9 ± 23 |
| Serum phosphate  (mmol/L) | 1.7 ± 0.1 | 1.8 ± 0.1 | | 1.6 ± 0.1 | | 0.1 ± 0.1 | | -0.1 ± 0.1 | 1.9 ± 0.1 | 1.8 ± 0.1 | 1.7 ± 0.1 | -0.0 ± 0.1 | -0.1 ± 0.1 |
| hsCRP (mg/L) | 3.0 ± 0.8 | 3.2 ± 0.8 | | 3.0 ± 0.8 | | 0.2 ± 0.2 | | 0.1 ± 0.2 | 4.3 ± 0.7 | 4.5 ± 0.8 | 4.2 ± 0.7 | 0.2 ± 0.3 | -0.1 ± 0.4 |
| IL-6 (pg/ml) | 4.2 ± 0.4 | 4.1 ± 0.4 | | 4.1 ± 0.4 | | -0.0 ± 0.2 | | -0.1 ± 0.3 | 4.1 ± 0.3 | 4.4 ± 0.4 | 4.4 ± 0.4 | 0.3 ± 0.2 | 0.3 ± 0.2 |
| *n*PCR (g/kg/day) | 1.0 ± 0.1 | 1.1 ± 0.1 | | 1.2 ± 0.1 | | 0.1 ± 0.1 | | 0.2 ± 0.1*^*^* | 1.0 ± 0.1 | 1.0 ± 0.1 | 1.0 ± 0.1 | 0.0 ± 0.1 | 0.0 ± 0.1 |
| ***Dietary parameters*** |  |  | |  | |  | |  |  |  |  |  |  |
| Energy (kcal)*^a,b,c^* | 1305 ± 58 | 1674 ± 55 | | 1671 ± 49 | | 369 ± 54*^**^* | | 366 ± 60*^**^* | 1313 ± 56 | 1373 ± 38 | 1389 ± 64 | 60 ± 35 | 76 ± 44 |
| Protein (g)*^a,b,c^* | 49.1 ± 3.4 | 64.9 ± 2.4 | | 66.5 ± 3.0 | | 15.8±2.4*^**^* | | 17.4±3.2*^**^* | 44.7 ± 2.7 | 47.8 ± 2.2 | 51.8 ± 3.6 | 3.1 ± 1.4 | 7.1 ± 2.4 |
| DEI (kcal/kg IBW) *^a,b,c^* | 24.8 ± 1.2 | 31.3 ± 1.3 | | 31.0 ± 1.1 | | 6.5 ± 1.0*^**^* | | 6.2 ± 1.2*^**^* | 24.2 ± 1.1 | 24.8 ± 0.9 | 25.7 ± 1.4 | 0.6 ± 0.8 | 1.5 ± 0.9 |
| DPI (g/kg IBW) *^a,b,c^* | 0.9 ± 0.1 | 1.2 ± 0.1 | | 1.2 ± 0.1 | | 0.3 ± 0.1*^**^* | | 0.3 ± 0.1*^**^* | 0.8 ± 0.1 | 0.9 ± 0.1 | 1.0 ± 0.1 | 0.1 ± 0.0 | 0.1 ± 0.1 |
| ***Appetite rating*** | | | | | | | | | | | | | |
| Good | 19 (65.5) | | 22 (75.9) | | 19 (65.5) | | ns | | 12 (44.4) | 19 (70.4) | 17 (63.0) | ns | |
| Diminished | 10 (34.5) | | 7 (24.1) | | 10 (34.5) | | ns | | 15 (55.6) | 8 (29.6) | 10 (37.0) | ns | |
| ***Physical activity level^ξ^*** | | | | | | | | | | | | | |
| PAL  (MET-minutes/week) | 198  (0-487) | 198  (0-644) | | 198  (17-747) | | 0  (-50-149) | | 0  (-221-289) | 198  (0-396) | 66  (0-347) | 66  (0-462) | 0  (-165-0) | 0  (-198-0) |

Note: All baseline comparisons between group were not significantly different as per Independent *t* test or Chi-square test; Data adjusted for age, gender, dialysis vintage and presence of diabetes mellitus is presented as mean ± standard deviation, median (interquartile range) or frequency (percentage); *^a^* Main effect of Group, *^b^* Main effect of Time, *^c^* Group x Time interaction; Δ*^t3^* =Mean change at 3^rd^ months, Δ*^t6^* =Mean change at 6^th^ months; *Significant at *p*<0.05 compared to baseline, ** Significant at *p*<0.001 compared to baseline

***^ξ^*** Logarithmic conversion was performed for PAL as data was non-normally distributed

Abbreviation: BMI, body mass index; DEI, dietary energy intake; DPI, dietary protein intake; hsCRP, high sensitivity C-reactive protein; IBW, ideal body weight; IL-6, interleukin-6; NC, nutritional counselling; *n*PCR, normalized protein catabolic rate; ONS, oral nutritional supplementation; PAL, physical activity level

**
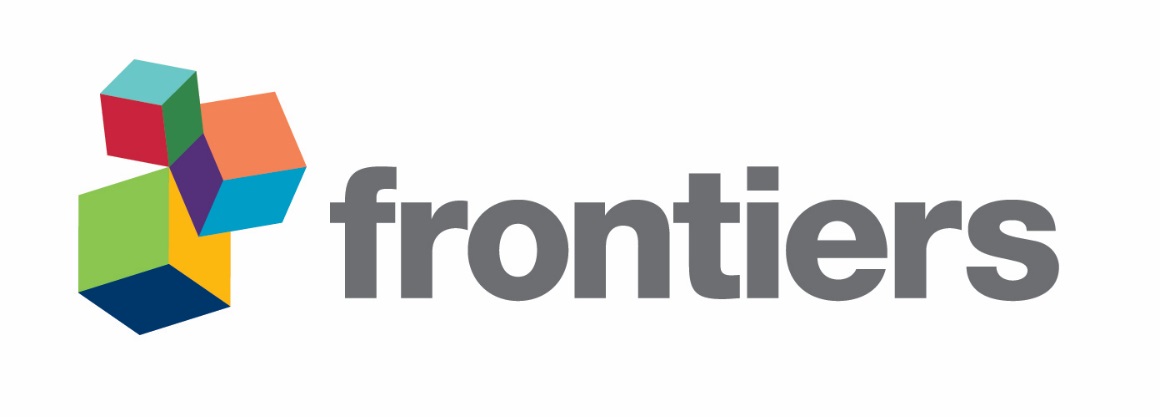
**
